# Herpes Zoster Oticus with Concurrent Hearing Loss: A Study on Clinical Characteristics and Prognosis

**DOI:** 10.3390/jcm12206476

**Published:** 2023-10-12

**Authors:** Hwa Sung Rim, Seok Hwan Chung, Ho Joong Kim, Seung Geun Yeo, Sang Hoon Kim

**Affiliations:** Department of Otolaryngology-Head & Neck Surgery, School of Medicine, Kyung Hee University, Seoul 130-701, Republic of Korea; marslover@naver.com (H.S.R.); seokhwanchung@naver.com (S.H.C.); feelingsky1@naver.com (H.J.K.); yeo2park@gmail.com (S.G.Y.)

**Keywords:** facial palsy, Herpes Zoster Oticus, hearing loss

## Abstract

This study aimed to analyze the clinical characteristics and treatment prognoses of patients with Herpes Zoster Oticus (HZO) and concurrent hearing loss (HL). Various clinical characteristics of 192 patients with HZO, with or without concurrent HL, from 2016 to 2020 were retrospectively analyzed through a chart review. All patients were followed-up until recovery or up to 12 months. Demographic and clinical findings were compared between the groups, and the recovery rates of facial palsy, hearing, and other clinical features were analyzed. Facial palsy recovery was analyzed using the House–Brackmann (HB) grading system, and hearing recovery rates were analyzed using the Siegel criteria. Of the 192 patients diagnosed with HZO, 142 had no hearing loss (HZO without HL), and 50 had hearing loss (HZO with HL). While both groups had similar ages, treatment timings, and underlying diseases, the HZO w HL group had a significantly higher rate of dizziness and tinnitus, but received more intratympanic steroid injections. In terms of facial palsy, there were no significant differences in the initial HB grade or recovery rates between the groups. Within the HZO w HL group, hearing loss severity varied, with 52% experiencing mild loss and only 16% achieving complete recovery. Descending-type audiograms were the most common at 66%. In patients with HZO, there was no statistically significant difference between the degree of initial facial paralysis and the degree of recovery of the final facial paralysis according to concurrent HL. However, among patients with concurrent HL, the hearing recovery rate in the HZO group was low.

## 1. Introduction

Herpes Zoster Oticus (HZO) is a disease characterized by facial nerve paralysis and otalgia, with concomitant small vesicular rashes in the external ear and auditory canal. It is caused by the reactivation of the latent varicella-zoster virus present in the geniculate ganglion [1]. The varicella-zoster virus characteristically invades the perineurium better than the herpes simplex virus, and can affect the facial, cochlear, and vestibular nerves, consequently causing sensorineural hearing loss and dizziness [2]. The symptoms of facial nerve palsy are more severe than those of Bell’s palsy, and the prognosis is poor [3].

The reactivation of this virus in the geniculate ganglion is thought to be triggered by factors such as aging, immunosuppression, or other stressors, and this reactivation leads to the clinical manifestation of HZO. Furthermore, the invasion of the varicella-zoster virus into the perineurium, a connective tissue sheath surrounding peripheral nerves, contributes to its distinct neuropathic effects. This invasion can result in inflammation, edema, and demyelination of the affected nerves, which subsequently leads to the development of sensorineural hearing loss and dizziness. The involvement of the facial, cochlear, and vestibular nerves in HZO underscores the complexity of the condition, as it affects both auditory and vestibular functions, often with a profound impact on a patient’s overall quality of life.

Hearing difficulties often appear in individuals with the Ramsay Hunt syndrome (RHS). Prior studies focusing on hearing challenges in patients with RHS have revealed that most patients experience a mild-to-moderate degree of hearing loss. This hearing impairment is commonly associated with the involvement of either the cochlear or retrocochlear pathways [4,5,6]. Anatomically, the facial and auditory nerves meet in the brain stem and in the inner ear canal, pass together, and enter the inner ear. Some also form anastomoses in the nerve stem and share the same vasculature; therefore, they can be affected by the same pathogenesis [7].

Given the interconnection of the facial and auditory nerves at both the level of the brain stem and within the inner ear canal, it is crucial to further elucidate the pathophysiological basis of hearing impairment in individuals afflicted with the Ramsay Hunt syndrome (RHS). The anatomical proximity and shared vasculature of these nerves raise intriguing questions about the mechanisms underlying auditory dysfunction in RHS. While previous research has provided valuable insights into the association between RHS and hearing loss, there remains a need for a deeper understanding of the exact processes that lead to hearing impairment. In addition to anatomical considerations, it is imperative to explore the potential role of viral pathogenesis in causing cochlear and retrocochlear hearing deficits in RHS. The reactivation of the varicella-zoster virus (VZV) in the geniculate ganglion, which is central to RHS, likely plays a pivotal role in the development of auditory symptoms. Investigating how VZV infiltrates and impacts the auditory nerves, particularly within the cochlear and retrocochlear pathways, is a fundamental step in comprehending the underlying pathology.

Moreover, research should also delve into the inflammatory and immunological processes that contribute to auditory nerve damage in RHS. Inflammation and immune responses triggered by VZV reactivation may induce structural and functional changes within the auditory system. A comprehensive examination of these immunopathogenic aspects is essential to paint a more complete picture of the complex interplay between viral infection, inflammation, and auditory dysfunction in RHS. By shedding light on these intricate processes, this research aims to enhance our understanding of the pathogenesis of hearing loss in RHS, potentially leading to the development of more effective diagnostic and therapeutic strategies. A comprehensive investigation of the anatomical, virological, and immunological aspects will provide a holistic perspective on the hearing challenges faced by RHS patients, ultimately improving the quality of care provided to this patient population. 

Several studies have been conducted on HZO and the symptoms associated with various cranial nerve invasions. However, no study has investigated the clinical characteristics of patients with HZO with or without hearing loss (HL), recovery from facial nerve palsy, or post-treatment hearing recovery in these patients. This study analyzed the clinical characteristics and treatment prognoses of patients with HZO, with or without HL.

## 2. Materials and Methods

### 2.1. Study Population

This study conducted a retrospective analysis on patients who were treated for Herpes Zoster Oticus (HZO), with or without hearing loss (HL), at the outpatient clinic or emergency room of our medical center from January 2016 to December 2020. Patients were identified through a meticulous review of medical records. A follow-up period of at least 12 months was ensured for all patients included in this study.

### 2.2. Inclusion and Exclusion Criteria

Patients were included if they had been diagnosed with Herpes Zoster Oticus. The exclusion criteria were as follows: pediatric patients, congenital malformations, trauma, infections other than HZO, otitis media, tumors, metabolic diseases, systemic diseases, autoimmune diseases, and Bell’s palsy as causes of facial paralysis. This exclusion helped in ascertaining the diagnosis of HZO by eliminating other possible causes of facial paralysis.

### 2.3. Diagnosis of Herpes Zoster Oticus

The diagnosis of Herpes Zoster Oticus was established based on a combination of clinical presentations, laboratory investigations, and, where applicable, radiological assessments. The following criteria were utilized to confirm the diagnosis of HZO:

 **A.** **Clinical Presentation**
  Patients presented with the characteristic triad of symptoms: otalgia, vesicular eruptions in the ear canal, and facial nerve paralysis. The presence of these clinical manifestations served as the primary indicators of HZO.
 **B.** **Laboratory Investigations**
  Polymerase Chain Reaction (PCR) Testing: PCR testing for Varicella Zoster Virus (VZV) DNA was conducted on samples obtained from vesicular lesions;
  Viral Culture: Additionally, viral culture of swabs taken from vesicular eruptions was performed to isolate and identify the VZV.
 **C.** **Radiological Assessments**
  In selected cases, radiological imaging, such as Magnetic Resonance Imaging (MRI) or Computed Tomography (CT) scans, was employed to assess the extent of cranial nerve involvement and exclude other potential causes of the symptoms observed.
 **D.** **Serological Testing**
  Serological assays for VZV antibodies were conducted to ascertain recent or active infection in patients with a less typical presentation or when PCR and culture results were equivocal.

Furthermore, the patients’ medical history, particularly past incidents of varicella infection or vaccination status, was also taken into consideration during the diagnostic process. The diagnostic criteria were employed stringently, ensuring a thorough and accurate confirmation of HZO in all the included patients. The multidisciplinary approach involving otolaryngologists, neurologists, and infectious disease specialists further ensured the accuracy and reliability of the diagnoses.

### 2.4. Treatment Protocols

All patients diagnosed with HZO received a combination of oral prednisolone and antiviral therapies. The prednisolone therapy was initiated at a dose of 1 mg/kg/day and was gradually tapered according to a standardized protocol. Famciclovir was administered at a dose of 750 mg/day for a duration of 7 days. Intratympanic steroid injections were administered when deemed necessary, twice a week at 3–4 day intervals, for a total of four times, with the aim of enabling outpatient treatment.

### 2.5. Outcome Assessments

Facial nerve function was assessed both initially and at the conclusion of the follow-up period by two experienced otologists employing the House–Brackmann (HB) facial nerve grading system [8]. Any discrepancies in grading were resolved by accepting the lower grading score as the final degree of facial palsy. Hearing levels were evaluated using the pure-tone audiometry by employing the six-division method. The audiogram patterns were further categorized as ascending, flat, or descending based on the hearing thresholds across different frequencies. Additional symptoms including tinnitus, dizziness, and hyperacusis were also investigated.

### 2.6. Statistical Analysis

Data analysis was carried out using IBM SPSS Statistics (version 24.0; IBM Corp., Armonk, NY, USA). The chi-square test, analysis of variance, and Fisher’s exact test were utilized for statistical analysis, as deemed appropriate, following a rigorous assessment of underlying assumptions. The level of statistical significance was set at *p* < 0.05.

### 2.7. Ethical Approval

The study protocol adhered to the ethical guidelines and was duly approved by our institutional review board (IRB No. 2019-07-065).

## 3. Results

Of the 192 included patients, 142 were diagnosed with HZO without HL (HZO w/o HL group; 76 men, 66 women) and 50 were diagnosed with HZO with HL (HZO w HL group; 27 men, 23 women). The mean age of patients in HZO w/o HL group, and HZO w HL group were 51.3 ± 19.2, 43.2 ± 13.1 years, respectively (*p* > 0.05). The laterality and treatment time after onset were similar between the two groups. In the HZO w/o HL group, 139 (97.8%) patients received oral steroid and antiviral agent treatment, and 3 (2.2%) patients received additional intratympanic steroid injections. In the HZO w HL group, 22 (44%) patients received oral steroid and antiviral agent treatments and 28 (56%) patients received additional intratympanic steroid injections. There was a significant difference in the number of patients who underwent intratympanic steroid injections between the two groups (*p* < 0.05), and there was no difference in underlying diseases, such as HTN and DM, between the groups. Compared with the HZO w/o HL group, the HZO w HL group had significantly higher rates of comorbid dizziness and tinnitus. However, there was no significant difference in the rate of hyperacusis between the two groups (Table 1).

When the initial facial palsy was classified into HB grades, in the HZO w/o HL group, HB grade V/VI facial palsy occurred in 70 (49.4%) patients, grade IV in 52 (36.6%), and grade II/III in 20 (14.0%). HZO w HL group had HB grade V/VI facial palsy in 26 (52.0%) patients, grade IV in 19 (38.0%), grade II/III in 5 (10.0%) patients. The initial HB grade was not significantly different between the HZO w/o HL and HZO w HL groups. When comparing the initial HB grades in patients with severe facial palsy (HB grade > IV), no statistically significant difference was observed between the two groups (HZO w/o HL group, 49.1%; HZO w HL group, 52.0%, *p* = 0.421). There was no significant difference in the ENoG results obtained within 14 days of the onset of paralysis between the HZO w/o HL and HZO w HL groups (*p* = 0.333). The CR rate (HB grade ≤ 1) of the final facial palsy was 68.3% in HZO w/o HL and 62% in HZO w HL, and there was no significant difference between the two groups (*p* = 0.233). When comparing the satisfactory recovery rate (HB grade ≤ 2), no statistically significant difference between the two groups (*p* = 0.233, 0.286) was found. History of facial palsy was noted in three patients (2.1%) in the HZO w/o HL group and one patient (2.0%) in HZO w HL group. Apart from the 7th and 8th cranial nerves, five patients (3.5%) in the HZO w/o HL group and four patients (8.0%) in the HZO w HL group had vocal cord paralysis or dysphagia associated with the 9th and 10th cranial nerves (Table 2).

On comparison of the initial hearing level in HZO w HL group, the rate of mild, moderate, moderately severe, severe, and profound hearing loss were 26 (52.0%), 10 (20.0%), 9 (18.0%), 3 (6.0%), and 2 (4.0%), respectively. The mean initial hearing thresholds was 51.4 ± 26.7 dB and the mean hearing threshold after treatment was 32.3 ± 17.9 dB. When divided according to Seigel’s criteria, complete recovery (CR) occurred in 8 (16.0%) patients, PR in 12 (24.0%), slight improvement (SI) in 7 (14.0%), and 23 patients (46.0%) showed no recovery. The rate of hearing recovery in the HZO with HL group was found to be lower compared to that of a general SSNHL population treated with high-dose steroids (66.7%) [9,10]. (Table 3).

**Figure 1:**
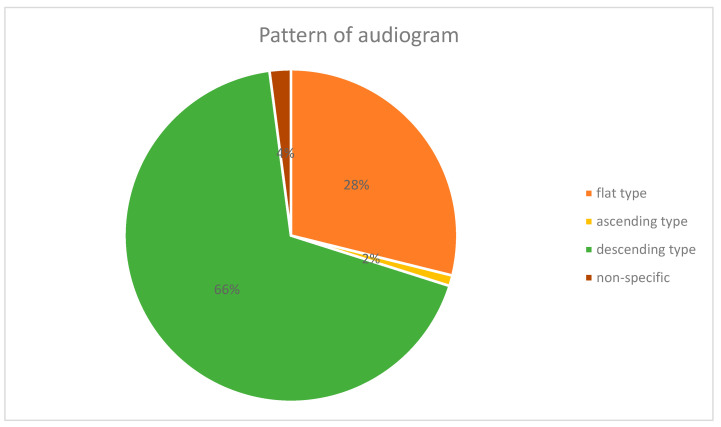
The pattern of audiograms in Herpes Zoster Oticus with hearing loss.

On comparison of the types of audiograms in the HZO w HL group, the descending type was the most frequent (33, 66.0%), followed by the flat type (14, 28.0%), and the non-specific type (2, 4%). The ascending type was the least common (1, 2.0%) (Figure 1).

## 4. Discussion

In the present investigation, we discovered that there was no significant difference in the degree of initial facial palsy or in the recovery of the final facial palsy between the HZO patients without HL and HZO patients with HL. Furthermore, the rate of hearing recovery in the HZO with HL group was observed to be lower than that in the SSNHL group. These outcomes are instrumental in providing better counseling for patients afflicted with HZO, and concurrent HL HZO is a viral syndrome that primarily manifests as herpes zoster lesions in the auricle, ear canal, and tympanic membrane, with otalgia [1]. HZO is the second most common cause of acute peripheral facial palsy, after Bell’s palsy. Among patients with unilateral facial palsy, the proportion with HZO was approximately 10%, and there was no specific difference between men and women. In the United States, the incidence rate of HZO is 5 per 100,000 people per year. It is more frequent in adults, especially the elderly, and rare in children [11]. HZO occurs more frequently in immunocompromised patients. In general, a higher prevalence of HZO is associated with a rapid decrease in immune function against herpes zoster virus after 60 years [12].

Herpes zoster virus, which causes HZO, invades the facial nerve following the vestibulocochlear nerve and can cause hearing loss, tinnitus, and vertigo. The pathological mechanism underlying these symptoms was inferred by detecting the varicella-zoster virus (VZV) genome in the facial nerve sheath, middle ear mucus, and cerebrospinal fluid, in addition to the vesicles of the auricle and oral cavity, as determined via polymerase chain reaction [13]. One possible pathway for VZV spread is from the facial nerve to the vestibulocochlear nerve through anastomosis of the nerve branches. The other is VZV dissemination to the oval and round windows through the inflow of viruses into the middle ear cavity via facial nerve defects. Conversely, Furuta et al. suggested that symptoms could be induced by the propagation of viral inflammation between nerves by detecting VZV in the spiral ganglion [14].

Because the cranial nerves in the brainstem, including the facial nerve, are located close to each other, symptoms can occur in the form of multiple cranial neuropathies. Inflammation can spread to the microvascular structure, which has a common distribution in other cranial nerves, and causes infarction [15]. Patients with multiple cranial neuropathies with severe symptoms showed complete recovery of facial nerve function within 3–5 days after treatment with a combination of steroid and antiviral therapy [16]. In other studies, the recovery rate from facial palsy was worse (54.5%) in patients with multiple cranial neuropathies than in those with simple Herpes Zoster Oticus (82.9%). The CR rate was lower in patients with multiple cranial neuropathies (27.3%) than in those with simple HZO (67.7%). These findings suggest that prognosis is poorer in patients with multiple cranial neuropathies, thus emphasizing the need for greater attention during treatment [17]. The involvement of microvascular structures that have a common distribution in several cranial nerves further complicates the clinical picture. In some cases, this vascular involvement can result in infarction, causing additional neurological deficits. This intricate interplay between inflammation and vascular compromise highlights the need for early and comprehensive medical intervention in patients with HZO, especially those exhibiting multiple cranial neuropathies. Moreover, the prognosis for patients with multiple cranial neuropathies in the context of HZO appears to be less favorable when compared to those with isolated facial nerve involvement. Research findings indicating a lower recovery rate for facial palsy and a reduced complete recovery rate in patients with multiple cranial neuropathies emphasize the challenges associated with managing this more complex subset of HZO cases. These observations underscore the significance of tailored treatment strategies for patients presenting with multiple cranial neuropathies, calling for a more vigilant approach in their care.

In this study, the occurrence rate of dizziness and tinnitus were higher in HZO w HL group, indicating that, in cases of facial palsy with HL, the spread of inflammation from the ganglion to the vestibulocochlear nerve due to VZV increases the rate of vestibulocochlear symptoms. Multiple cranial neuropathies was observed in five (3.5%) patients in the HZO w/o HL group and in four (8.0%) patients in the HZO w HL group, which was not significantly different from that reported in previous studies.

Oral steroid therapy has been mainly used to promote the disappearance of vesicles, reduce swelling to relieve pain in the acute phase, reduce the occurrence of postherpetic neuralgia, and improve the recovery rate of facial palsy. The development of antiviral drugs that inhibit viral replication has promoted their widespread use. No randomized controlled study has compared the efficacy of antiviral agents either alone or in combination with adjuvant steroid therapy; however, it cannot be assumed that antiviral agents are ineffective even if evidence-based effects have not been proven [18]. Combined steroid and antiviral therapies continue to be considered as the most effective treatments.

HZO is a disease that requires prompt medical attention. It is well known that the faster the treatment, the better the prognosis. According to the results of several studies on HZO, if both steroids and antiviral agents are administered promptly, the overall outcomes improve. In the group treated within 3 days of onset, the CR of facial function reached 75% compared to the group that started treatment after 7 days, where it dropped to 30% [19]. This study was conducted on patients who started treatment within 7 days to reduce prognostic bias according to the treatment period between each group. Finally, 97 patients (68.3%) in HZO w/o HL and 31 (62.0%) in HZO w HL showed satisfactory recovery, and there were no differences in the recovery rate of facial palsy between the two groups. In previous studies, the incidence of vestibular dysfunction in patients with Herpes Zoster Oticus increased as severity of facial paralysis increased, while that of cochlear dysfunction did not [4]. Our findings resonate with these studies.

Among the patients with Herpes Zoster Oticus (HZO) exhibiting vestibulocochlear symptoms, various degrees of sensorineural hearing loss (SNHL) in the ipsilateral ear were identified upon otological examination. In the auditory brainstem response test, abnormal findings such as no response, prolongation of the III and V wave latencies, and increases in the I–III latency and I–V latency were observed. The results of transient-evoked otoacoustic emission were also abnormal, indicating the presence of various types of hearing loss, such as labyrinthine, post-labyrinthine, and mixed type [20].

In a retrospective study of 111 patients with HZO conducted by Kim et al., pure-tone audiometry (PTA) results showed abnormal findings in 79% of patients with initial audiologic symptoms and 56% of patients without any audiologic symptoms. Therefore, they insisted that PTA should be conducted. However, no relationship was found between the severity of facial palsy and the presence of auditory symptoms. Hearing impairment was more prominent in the high-frequency group. Abnormal vestibular function in patients with dizziness is proportional to the severity of the facial palsy [4].

In this study, when comparing the audiogram patterns in the HZO and HL groups, the ratio of the descending type was significantly higher in the HZO w HL group, which was consistent with the results of previous studies [4,21]. 

Mia et al. reported that magnetic resonance imaging (MRI) showed enhancement of the cochlear nerve in the inner auditory canal of HZO patients with unrecovered SNHL [22]. This finding supports the hypothesis that VZV activation causes cochlear neuritis in patients with HZO, resulting in refractory SNHL. Wayman et al. reported that older patients with HZO who had vertigo and retrocochlear hearing loss showed poorer hearing recovery rates [6]. In this study, the prognosis of hearing loss in the HZO w HL group was worse than the overall recovery rate in the SSNHL group with mild-to-moderate HL [23]. HL caused by VZV may affect the inner ear, cochlea, and microvascular structures extensively, and the prognosis of HL may decrease. On comparing the treatments, it was observed that intratympanic steroid injections were administered more frequently in the HZO w HL group. In most patients in the HZO w HL group, the recovery of HL after oral steroid treatment was insufficient, indicating the need for additional treatment.

This study has several limitations. First, this was a retrospective review of the medical records. MRI and hearing function tests were not performed in any of the patients included in this study. Secondly, cochlear, or retrocochlear involvement could not be identified because ABR and OAE were not conducted in the patients with hearing loss. Third, since all tests were not performed in patients with dizziness, they were excluded from the analysis.

As this study was a retrospective analysis, and the number of patients was insufficient, a multicenter or large-scale prospective study is needed to analyze the correlations and prognostic factors of hearing and other related symptoms in patients with HZO. In future studies, more objective results should be obtained through electrophysiological tests, such as ABR, for additional auditory functions in all patients.

## 5. Conclusions

In this study, no significant difference was observed in the degree of initial facial palsy or the recovery of final facial palsy between HZO patients without HL and those with HL. However, we noted a lower rate of hearing recovery in the HZO with HL group when compared to a general population of SSNHL patients treated with high-dose steroids. This comparative observation underpins the potential impact of early high-dose steroid treatment in enhancing hearing recovery rates in SSNHL cases, providing valuable insights for better patient counseling in scenarios of HZO concurrent with HL.

## Figures and Tables

**Table 1:** Patient characteristics.

	HZO w/o HL (*n* = 142)	HZO w HL (*n* = 50)	*p*-Value
Age	51.3 ± 19.2	43.2 ± 13.1	0.211
Male/Female	76/66	27/23	0.273
Right/Left	68/74	24/26	0.423
Treatment time after onset (days)	3 ± 1.4	2 ± 2.9	0.219
Treatment			
Oral steroid	139 (97.8)	22 (44.0%)	*p* < 0.05
Oral steroid+ ITDx	3 (2.2%)	28 (56.0%)	*p* < 0.05
Underlying disease			
HTN	39 (27.5%)	12 (24.0%)	0.425
DM	19 (13.4%)	10 (20.0%)	0.276
Accompanying symptoms			
Dizziness	62 (43.6%)	32 (64%)	*p* < 0.05
Tinnitus	9 (6.3%)	31 (62%)	*p* < 0.05
Hyperacusis	27 (19.0%)	10 (20%)	0.312

HZO, Herpes Zoster Oticus; HL, Hearing loss; ITDx, Intratympanic Dexamethasone injection; DM, Diabetes Mellitus; HTN, Hypertension.

**Table 2:** Comparison of clinical data related to facial paralysis.

	HZO w/o HL (*n* = 142)	HZO w HL (*n* = 50)	*p*-Value
Initial HB grade			
II/III	20 (14.0%)	5 (10.0%)	0.323
IV	52 (36.6%)	19 (38.0%)	0.578
V/VI	70 (49.4%)	26 (52.0%)	0.421
ENoG (%)	75.5 ± 25.1	80.1 ± 17.3	0.333
Final HB grade			
I	97 (68.3%)	31 (62.0%)	0.233
II	23 (16.1%)	10 (20.0%)	0.286
III	18 (12.6%)	6 (12.0%)	0.235
IV	4 (2.8%)	3 (6.0%)	0.114
Previous facial palsy history	3 (2.1%)	1 (2.0%)	
Cranial polyneuropathy (exclude CN VII or VIII)	62 (43.6%)	32 (64%)	*p* < 0.05

HZO, Herpes Zoster Oticus; HL, Hearing loss; ENoG, Electroneurography; HB, House–Brackmann; CN, cranial nerve.

**Table 3:** Hearing recovery rate of Herpes Zoster Oticus with hearing loss.

Post-Treatment Hearing Recovery Outcomes						
	CR	PR	SI	NI	Hearing Improvement: (CR + PR + SI)/Subtotal	Complete Hearing Improvement: CR/Subtotal
Pre-Treatment Hearing Level						
26–40 dB	6	8	2	10	16/26 (61.5%)	6/26 (23.1%)
40–45 dB	1	3	2	4	6/10(60%)	1/10 (10.0%)
45–70 dB	1	1	2	5	4/9 (44.4%)	1/9 (11.1%)
70–90 dB	0	0	1	2	1/3(33.3%	0/3 (0%)
90<	0	0	0	2	0/2 (5%)	0/2 (0%)
total	8	12	7	23	27/50 (54%)	8/50 (16%)

CR, complete recovery; PR: partial recovery; SI: slight improvement; NI, no improvement.

## Data Availability

Data were collected during the study in Kyunghee Medical Center.

## References

[1] Sweeney C., Gilden D. (2001). Ramsay hunt syndrome. J. Neurol. Neurosurg. Psychiatry.

[2] Niesvizky-Kogan I., Greca I., Gambhir H.S. (2019). Varicella zoster presenting as cranial polyneuropathy. Am. J. Emerg. Med..

[3] Kim S.H., Jung J., Jung S.Y., Dong S.H., Byun J.Y., Park M.S., Kim S.H., Yeo S.G. (2019). Comparative prognosis in patients with Ramsay-Hunt syndrome and Bell’s palsy. Eur. Arch. Oto-Rhino-Laryngol..

[4] Kim J., Jung J., Moon I.S., Lee H.-K., Lee W.-S. (2008). Statistical analysis of pure tone audiometry and caloric test in herpes zoster oticus. Clin. Exp. Otorhinolaryngol..

[5] Kuhweide R., Van de Steene V., Vlaminck S., Casselman J. (2002). Ramsay Hunt syndrome: Pathophysiology of cochleovestibular symptoms. J. Laryngol. Otol..

[6] Wayman D., Pham H., Byl F., Adour K. (1990). Audiological manifestations of Ramsay Hunt syndrome. J. Laryngol. Otol..

[7] Özdoğmuş Ö., Sezen O., Kubilay U., Saka E., Duman U., Şan T., Çavdar S. (2004). Connections between the facial, vestibular and cochlear nerve bundles within the internal auditory canal. J. Anat..

[8] House J.W., Brackmann D.E. (1985). Facial nerve grading system. Otolaryngol.—Head Neck Surg..

[9] Lin H.C., Wang C.H., Chou Y.C., Shih C.P., Chu Y.H., Lee J.C., Chen H.C. (2015). The correlation between lipoprotein ratios and hearing outcome in idiopathic sudden sensorineural hearing loss patients. Clin. Otolaryngol..

[10] Chen W.T., Lee J.W., Yuan C.H., Chen R.F. (2015). Oral steroid treatment for idiopathic sudden sensorineural hearing loss. Saudi Med. J..

[11] Cai Z., Li H., Wang X., Niu X., Ni P., Zhang W., Shao B. (2017). Prognostic factors of Bell’s palsy and Ramsay Hunt syndrome. Medicine.

[12] Goldani L., Ferreira da Silva L., Dora J. (2009). Ramsay Hunt syndrome in patients infected with human immunodeficiency virus. Clin. Exp. Dermatol..

[13] Murakami S., Nakashiro Y., Mizobuchi M., Hato N., Honda N., Gyo K. (1998). Varicella-zoster virus distribution in Ramsay Hunt syndrome revealed by polymerase chain reaction. Acta Oto-Laryngol..

[14] Furuta Y., Takasu T., Fukuda S., Sato-Matsumura K.C., Inuyama Y., Hondo R., Nagashima K. (1992). Detection of varicella-zoster virus DNA in human geniculate ganglia by polymerase chain reaction. J. Infect. Dis..

[15] Gilden D., Cohrs R.J., Mahalingam R., Nagel M.A. (2009). Varicella zoster virus vasculopathies: Diverse clinical manifestations, laboratory features, pathogenesis, and treatment. Lancet Neurol..

[16] Kim Y.H., Chang M.Y., Jung H.H., Park Y.S., Lee S.H., Lee J.H., Oh S.H., Chang S.O., Koo J.W. (2010). Prognosis of Ramsay Hunt syndrome presenting as cranial polyneuropathy. Laryngoscope.

[17] Shim H.J., Jung H., Park D.C., Lee J.H., Yeo S.G. (2011). Ramsay Hunt syndrome with multicranial nerve involvement. Acta Oto-Laryngol..

[18] Uscategui T., Doree C., Chamberlain I.J., Burton M.J. (2008). Antiviral therapy for Ramsay Hunt syndrome (herpes zoster oticus with facial palsy) in adults. Cochrane Database Syst. Rev..

[19] Minakata T., Inagaki A., Sekiya S., Murakami S. (2019). Contrast-enhanced magnetic resonance imaging of facial nerve swelling in patients with severe Ramsay Hunt syndrome. Auris Nasus Larynx.

[20] Hato N., Kisaki H., Honda N., Gyo K., Murakami S., Yanagihara N. (2000). Ramsay Hunt syndrome in children. Ann. Neurol. Off. J. Am. Neurol. Assoc. Child Neurol. Soc..

[21] Kim C.-H., Choi H., Shin J.E. (2016). Characteristics of hearing loss in patients with herpes zoster oticus. Medicine.

[22] Takahashi M., Sato G., Toda N., Azuma T., Nakamura K., Iwasaki H., Miyoshi H., Matsuda K., Kitamura Y., Abe K. (2021). Vestibular and cochlear nerve enhancement on MRI and its correlation with vestibulocochlear functional deficits in patients with Ramsay Hunt syndrome. Auris Nasus Larynx.

[23] Chandrasekhar S.S., Tsai Do B.S., Schwartz S.R., Bontempo L.J., Faucett E.A., Finestone S.A., Hollingsworth D.B., Kelley D.M., Kmucha S.T., Moonis G. (2019). Clinical practice guideline: Sudden hearing loss (update). Otolaryngol.–Head Neck Surg..

